# Uma Nova Fórmula Corrigida para Estimar Corretamente a Pressão Aórtica Central a Partir de Medições Periféricas da Braçadeira

**DOI:** 10.36660/abc.20240880

**Published:** 2025-09-08

**Authors:** Mehmet Ozgeyik, Onur Kaypakli

**Affiliations:** 1 Eskisehir City Hospital Department of Cardiology Eskisehir Turquia Eskisehir City Hospital, Department of Cardiology, Eskisehir – Turquia; 2 Hatay University Faculty of Medicine Department of Cardiology Hatay Turquia Hatay University, Faculty of Medicine, Department of Cardiology, Hatay – Turquia

**Keywords:** Pressão Arterial, Pulso Arterial, Palpação

## Abstract

**Fundamento:**

A pressão arterial média (PAM) tem importância crucial na perfusão tecidual. Na prática clínica, a fórmula mais utilizada foi sugerida por Gauer, que utiliza as pressões sistólica (PAS), diastólica (PAD) e de pulso (PP) coletadas pela artéria ilíaca (PAM = PAD + 0,333 x PP). No entanto, seus resultados não são confiáveis para registros não invasivos, pois as pressões arteriais são mais elevadas.

**Objetivos:**

Derivamos uma fórmula corrigida para o cálculo correto da PAM a partir dos registros de pressão arterial não invasiva: PAM = PADmanguito + [0,33 + (0,43 – 0,0038 x PADmanguito)] x PPmanguito.

**Métodos:**

149 pacientes foram incluídos neste estudo. Os traçados da pressão arterial intra-aórtica e do manguito foram obtidos simultaneamente. O coeficiente PP da fórmula padrão é 0,333 para todos os cálculos. O desvio do coeficiente PP da fórmula padrão foi calculado com a fórmula do coeficiente PP −0,333. Essas duas fórmulas foram comparadas usando análise de regressão linear e o critério de informação de Akaike (AIC). O nível de significância adotado na análise estatística foi de 5%.

**Resultados:**

A pressão intra-aórtica média medida foi de 111,5 ± 13,0 mmHg. A pressão intra-aórtica média calculada pela fórmula padrão e pela fórmula corrigida foi de 105,8 ± 13,5 e 111,3 ± 12,1, respectivamente. O R, R^2^ e AIC da fórmula corrigida foram melhores do que os da fórmula padrão [(0,905 vs. 0,887), (0,818 vs. 0,787) e (858,9 vs. 1002,7), respectivamente].

**Conclusão:**

Até onde sabemos, este é o primeiro estudo para o cálculo da PAM a partir de medidas do pulso, e a fórmula corrigida tem melhor precisão do que a fórmula padrão para estimativa da PAM.

## Graphical abstract

**Figura Central:**
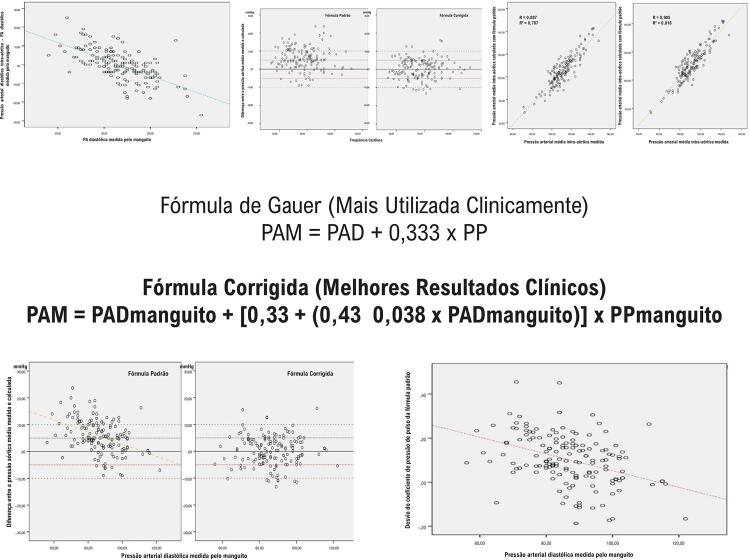
Uma Nova Fórmula Corrigida para Estimar Corretamente a Pressão Aórtica Central a Partir de Medições Periféricas da Braçadeira

## Introdução

A perfusão tecidual é muito importante para manter as funções vitais do corpo. A pressão arterial média (PAM) tem importância crucial na perfusão tecidual. Valores mais altos e mais baixos de PAM afetam negativamente os mecanismos celulares. Portanto, o corpo deve garantir uma PAM estável, e o cálculo correto da PAM pode salvar vidas em situações críticas.

O cálculo da pressão arterial central média exata é obtido a partir da pressão intra-aórtica. A área sob a curva da forma de onda pressão-tempo de um ciclo cardíaco completo com a integral ponderada no tempo é a maneira correta de calcular a pressão arterial central média.^1^ Cálculos da PAM por diferentes fórmulas foram sugeridas até agora. A primeira fórmula sugerida por Gauer usa pressões sistólica (PAS), diastólica (PAD) e de pulso (PP) coletadas pela artéria ilíaca (PAM=PAD+0,333×PP) .

Além disso, os estudos seguintes mostraram que essa fórmula tinha baixa precisão e adicionaram parâmetros como frequência cardíaca (FC) para obter fórmulas mais corretas.^3-5^ Por fim, Kaypakli et al. compararam todas as fórmulas e sugeriram uma fórmula melhor para o cálculo da PAM (PAM=PAD+[0,33+(0,16−0,00107×PP)+(−0,06+0,000773×FC)]×PP) .^6^ No entanto, a obtenção de valores de pressão arterial intra-aórtica geralmente não é viável na prática clínica diária.

Na prática clínica, os parâmetros de pressão arterial do esfigmomanômetro de manguito são utilizados para o cálculo da PAM. A fórmula de Gauer é geralmente utilizada para o cálculo da PAM, pois é fácil de usar. No entanto, esta fórmula empírica não foi comparada com uma fórmula melhor anteriormente. Neste estudo, derivamos uma fórmula corrigida para o cálculo da pressão arterial central média a partir dos registros da pressão arterial do manguito:  Pressão aórtica média = PAD manguito +[0,33+(0,43−0,0038x PAD manguito )]× PP manguito  Até onde sabemos, este é o primeiro estudo a calcular a pressão aórtica média a partir de medições do manguito.

## Materiais e métodos

Cento e quarenta e nove pacientes (70 homens e 79 mulheres) submetidos à angiografia coronária eletiva foram incluídos neste estudo. Foram incluídos todos os pacientes que atendiam aos critérios de exclusão entre maio de 2023 e dezembro de 2023 (*All-comers design*). O tamanho da amostra não foi determinado no início do estudo. Pacientes com fração de ejeção do ventrículo esquerdo <50%, doenças valvares leves ou graves, desequilíbrio eletrolítico, distúrbios do ritmo, síndromes coronárias agudas e menores de 18 anos foram excluídos do estudo. Todos os pacientes forneceram consentimento informado por escrito, e nosso Comitê de Ética Local aprovou o estudo.

O histórico médico, os parâmetros sanguíneos e as características basais foram obtidos a partir de registros hospitalares. O mesmo cardiologista realizou visualizações ecocardiográficas antes das angiografias. A fração de ejeção do ventrículo esquerdo foi calculada pela equação de Simpson.

Todas as angiografias coronárias foram realizadas pela artéria femoral. Cateteres diagnósticos 6 F (*Pig-tail*) foram inseridos na raiz da aorta para cálculo das pressões aórticas médias. A PAM foi calculada pelo método da curva de área sob pressão-tempo. Um dispositivo oscilométrico automatizado padrão (Bosomat, Bosooscillomat, Bosch, Jungingen, Alemanha, tamanho da bexiga 28 x 12,5) foi usado para medições da pressão arterial periférica. Os traçados da pressão arterial intra-aórtica e do manguito foram obtidos simultaneamente. Durante as aferições, todos os pacientes estavam em ritmo sinusal. Todas as aferições foram obtidas pelo mesmo cardiologista.

### Análises estatísticas

O teste de Kolmogorov-Smirnov foi realizado para verificar se as variáveis apresentavam distribuição normal, e um valor de p > 0,05 foi definido como dados com distribuição normal. Dados categóricos e contínuos foram expressos como porcentagem (%) e média ± desvio padrão (DP), respectivamente. A correlação de Pearson foi utilizada para examinar a relação entre variáveis contínuas. O IBM SPSS Statistics para Windows v. 23 foi utilizado para análises estatísticas, e valores de p < 0,05 foram considerados estatisticamente significativos.

Calculamos o coeficiente PP para valores medidos com a fórmula de (PAM medido-PAD)/PP . Como o coeficiente PP da fórmula padrão é 0,333 para todos os cálculos, o desvio do coeficiente PP da fórmula padrão foi calculado com a fórmula do coeficiente PP − 0,333. Em seguida, determinamos as correlações do desvio do coeficiente PP da fórmula padrão com variáveis clínicas contínuas. Como decidimos que a PAD das medições do manguito está mais correlacionada com o desvio do coeficiente PP da fórmula padrão (R: -,393, p < 0,001), realizamos uma análise de gráfico de dispersão do desvio do coeficiente PP da fórmula padrão com a PAD das medições do manguito. Neste gráfico de dispersão, reunimos esta equação:  desvio do coeficiente PP=0,43−0,0038x PAD do manguito  Depois, adicionamos esta equação à fórmula original, que é PAM=PAD+0,33×PP . Portanto, concluímos com esta fórmula:  Pressão aórtica média = PAD  manguito +[0,33+(0,43−0,0038× PAD manguito )]× PP manguito  As diferenças entre a PAM medida e a PAM calculada foram determinadas em cada ponto de medição e foram usadas para avaliar a precisão das duas fórmulas diferentes: a fórmula padrão (PAM=PAD+(0,33×PP) e a fórmula corrigida ( PAMaórtica = PADmanguito +[0,33+(0,43-0,0038x PADmanguito)]xPP) . Primeiramente, usamos o método gráfico descrito por Bland e Altman.^7^ Calculamos R, R^2^, resíduos quadrados médios (MSR = soma dos quadrados resíduos/n) e a raiz quadrada do erro quadrático médio (RMSE = √MSR) usando análise de regressão linear. Todas as seis suposições necessárias para o uso da análise de regressão linear foram verificadas. Como valores maiores de R e R^2^ indicam maior precisão, eles indicam concordância teórica perfeita quando são iguais a 1. Valores menores de RMSE e MSR indicam maior precisão; indicam concordância teórica perfeita quando são iguais a 0. Também obtivemos o critério de informação de Akaike (AIC) a partir de modelos lineares generalizados usando a fórmula AIC=N×ln⁡(RSS)+2P .

O AIC fornece um valor matemático para a avaliação de diferentes métodos de cálculo. Valores menores indicam melhor precisão. Finalmente, testamos a precisão das quatro fórmulas usando análise de regressão linear multivariada.

## Resultados

**Figura 1:**
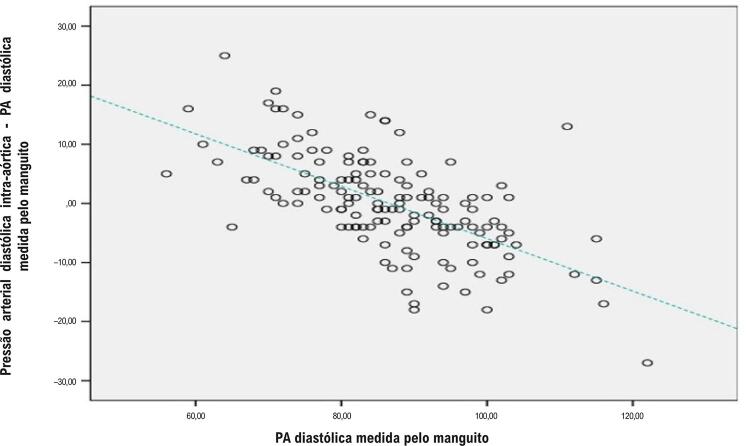
– Correlação entre a PA diastólica medida pelo manguito e a diferença entre a PA diastólica intra-aórtica e a PA diastólica medida pelo manguito.

**Figura 2:**
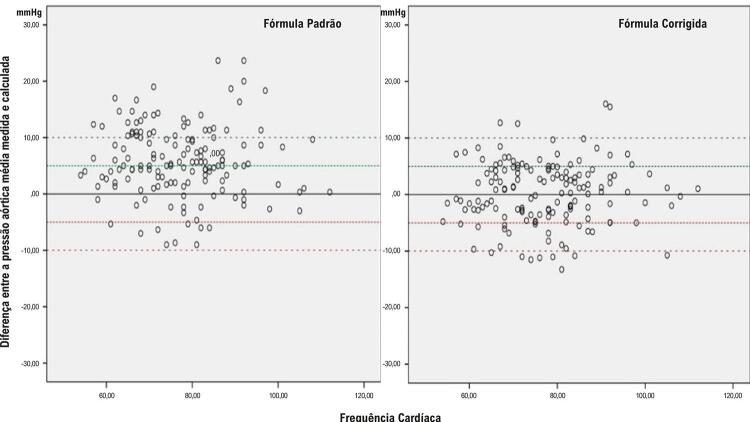
– Diferenças entre as pressões aórticas médias medidas e calculadas de acordo com a frequência cardíaca para a fórmula padrão e a fórmula corrigida.

**Figura 3:**
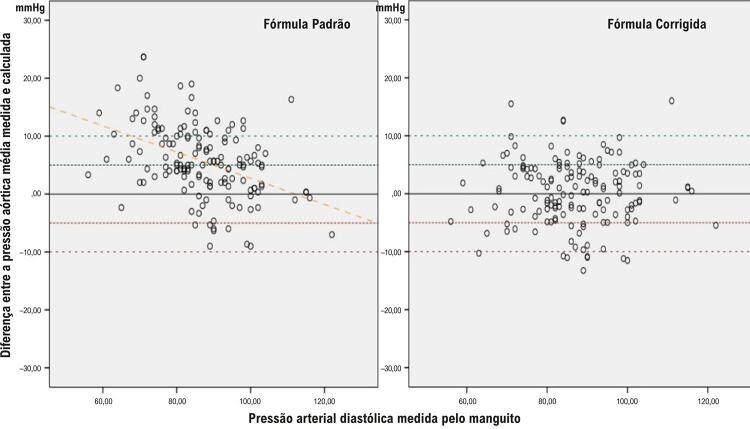
– Correlação entre as diferenças entre a pressão aórtica média medida e calculada e as pressões arteriais diastólicas medidas pelo manguito para fórmulas padrão e calculadas.

Houve um total de 298 pontos de medição da pressão arterial de 149 pacientes diferentes (149 medições intra-aórticas e 149 medições simultâneas do manguito). As informações sobre as características basais da população do estudo são mostradas na Tabela 1. A pressão média intra-aórtica medida foi de 111,5 ± 13,0 mmHg. A pressão média intra-aórtica calculada pela fórmula padrão e pela fórmula corrigida foi de 105,8 ± 13,5 e 111,3 ± 12,1, respectivamente. A Figura 1 mostra a correlação negativa entre a PA diastólica medida pelo manguito e a diferença entre a PA diastólica intra-aórtica e a PA diastólica medida pelo manguito. Este resultado nos mostra que as medições diastólicas do manguito aumentam mais do que as medições aórticas em valores diastólicos altos. As diferenças entre as pressões aórticas médias medidas e calculadas, conforme descrito por Bland e Altman^7^ para a fórmula padrão e a fórmula corrigida, foram demonstradas na Figura 2. A Figura 3 mostra as correlações entre as diferenças entre a pressão aórtica média medida e calculada e as pressões arteriais diastólicas medidas pelo manguito para as fórmulas padrão e calculada. Este resultado indica que a fórmula corrigida funciona melhor do que a fórmula padrão em valores de PAD mais elevados (Figura Central).

**Tabela 1:** – Características clínicas e demográficas basais

Variáveis	N=149
Idade (anos)	55,2 ± 10,3
Sexo Masculino,%)	70 (47,0)
Diabetes (n,%)	50 (33,6)
Hipertensão (n,%)	87 (58,4)
Tabagismo (n,%)	50 (33,6)
Histórico de DAC (n,%)	18 (12.1)
Hiperlipidemia (n,%)	47 (31,5)
Hemoglobina (g/dl)	13,8 ± 1,8
Creatinina (mg/dl)	0,83 ± 0,17
LDL (mg/dl)	111,9 ± 36,1
PAS intra-aórtica (mmHg)	145,6 ± 22,7
PAD intra-aórtica (mmHg)	86,6 ± 9,2
PAS medida pelo manguito (mmHg)	143,9 ± 21,0
PAD medida pelo manguito (mmHg)	86,7 ± 12,0
Pressão de pulso medida pelo manguito (mmHg)	57,2 ± 16,4
Pressão média intraaórtica medida (mmHg)	111,5± 13,0
Pressão média intra-aórtica calculada (fórmula padrão) (mmHg)	105,8 ± 13,5
Pressão média intra-aórtica calculada (fórmula corrigida) (mmHg)	111,3 ± 12,1
Frequência cardíaca (batimentos/min)	77,2 ± 11,8
Fração de Ejeção	58,9± 3,6
Betabloqueadores (n,%)	43 (28,9)
IECA ou BRA (n,%)	60 (40,3)
Bloqueador dos canais de cálcio (n,%)	25(16,8)
Diurético (n,%)	44 (29,5)

LDL: lipoproteína de baixa densidade; IECA: inibidores da enzima conversora de angiotensina; BRA: bloqueadores dos receptores da angiotensina; DAC: doença arterial coronariana; PAS: pressão arterial sistólica; PAD: pressão arterial diastólica.

**Figura 4:**
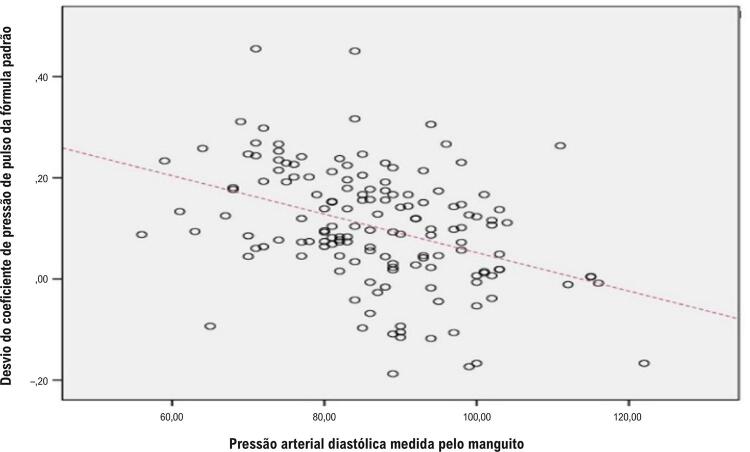
– Correlação entre o desvio do coeficiente PP da fórmula padrão e a pressão arterial diastólica medida pelo manguito.

A análise de correlação bivariada de variáveis clínicas contínuas com desvio do coeficiente de pressão de pulso da fórmula padrão é mostrada na Tabela 2. Além disso, a Figura 4 demonstra uma correlação positiva entre o desvio do coeficiente PP da fórmula padrão e a PAD medida pelo manguito.

**Tabela 2:** – Análise de correlação bivariada de variáveis clínicas contínuas com desvio do coeficiente de pressão de pulso da fórmula padrão

Variáveis	r	p
Frequência cardíaca	-0,079	0,337
Pressão arterial sistólica	-0,285	< 0,001
Pressão arterial diastólica	-0,393	< 0,001
Pressão de pulso	-0,078	0,344
Idade	-0,174	0,035
Fração de Ejeção	0,048	0,579

Coeficiente de pressão de pulso: (Pressão arterial média intra-aórtica - pressão arterial diastólica do manguito) / Pressão de pulso do manguito. Desvio do coeficiente de pressão de pulso da fórmula padrão: Coeficiente de pressão de pulso - 0,33.

**Figura 5:**
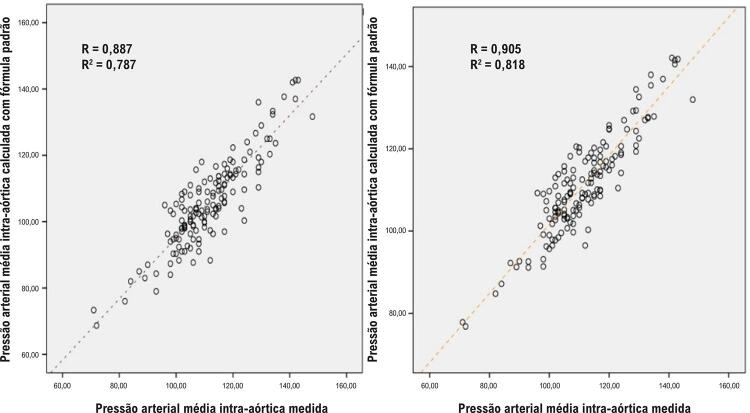
– Comparação da pressão arterial média intra-aórtica da fórmula padrão e da fórmula corrigida segundo R e R2.

Os parâmetros de precisão para prever a pressão arterial central média são mostrados na Tabela 3. Embora valores mais altos de R e R^2^ indiquem maior precisão, valores mais baixos de RMSE e MSR mostram maior precisão. O R da fórmula corrigida (0,905) foi melhor do que a fórmula padrão (0,887). O R^2^ da fórmula corrigida (0,818) foi melhor do que a fórmula padrão (0,787) (Figura 5). O RMSE e o MSR da fórmula corrigida foram 5,577 mmHg e 31,104 mmHg^2^, respectivamente, que foram melhores do que a fórmula padrão, que foi de 6,042 mmHg e 36,506 mmHg^2^, respectivamente. Valores mais baixos de AIC indicam melhor precisão. O valor de AIC da fórmula corrigida (858,9) foi superior ao da fórmula padrão (1002,7).

**Tabela 3:** – Comparação dos parâmetros de precisão de diferentes fórmulas para predizer a pressão arterial média

Parâmetro de precisão	Fórmula padrão	Fórmula corrigida
R	0,887	0,905
R^2^	0,787	0,818
Resíduos quadrados médios (mmHg^2^)	36.506	31.104
Erro quadrático médio (mmHg)	6.042	5.577
Critério de informação de Akaike (AIC)	1002.746	858.912

A pressão arterial central média medida foi prevista de forma independente apenas pela fórmula corrigida na análise de regressão linear multivariada (beta = 0,975, p < 0,001). A Tabela 4 mostra a análise de regressão linear multivariada.

**Tabela 4:** – Análise de regressão linear multivariada de duas fórmulas diferentes para prever a pressão aórtica média medida

	Beta	IC de 95% para Beta	p
Fórmula corrigida	0,975	0,901 -1,050	<0,001
Fórmula Padrão	0,097	-0,205 - 0,398	0,528

## Discussão

Até onde sabemos, este é o primeiro estudo a calcular a pressão aórtica média a partir de medições do manguito. A principal descoberta deste estudo é que, quando comparada à fórmula padrão de Gauer,^2^ a nova fórmula corrigida se mostrou mais precisa do que a fórmula padrão em termos de todos os critérios de precisão.

O aumento da pressão arterial está relacionado à morbidade e mortalidade cardiovascular.^9^ Especialmente, a pressão aórtica central (PAC) prediz eventos cardiovasculares mais do que a pressão arterial periférica.^10^ Em contraste com o aumento da PAC, na situação de sepse e choque, a PAM tem uma importância crítica para estimar danos em órgãos-alvo.^11,12^ A PAM é almejada para estar acima de 65 mmHg para reduzir a falência de órgãos em choque séptico; no entanto, o cálculo depende da canulação arterial central ou radial. Portanto, a estimativa da PAC com técnicas não invasivas pode ser muito benéfica para os médicos em termos do processo de tomada de decisão, especialmente para prever a perfusão de órgãos em pacientes com condições críticas, como dissecção aórtica, sepse e choque.^13-15^ No entanto, o cálculo da PAC é mais difícil e caro do que as medidas do manguito e precisa de uma abordagem invasiva. Muitas fórmulas foram criadas para descobrir o cálculo de PAM mais preciso.^2-6^ No entanto, essas fórmulas foram produzidas usando os valores da pressão aórtica central. Até onde sabemos, este é o primeiro estudo para o cálculo da pressão aórtica média a partir de medidas do manguito.

A fórmula corrigida é comparada com a fórmula padrão. A fórmula corrigida é superior à fórmula padrão em todos os parâmetros de precisão de AIC, R, R^2^, MSR e RMSE. Na análise de regressão linear multivariada, a nova fórmula corrigida prevê a pressão aórtica média de forma independente, ao contrário da fórmula padrão. Todas essas análises estatísticas mostraram que a fórmula corrigida calcula a pressão aórtica média com mais precisão do que a fórmula padrão.

A pressão arterial periférica é afetada não apenas pelo volume sistólico cardíaco, mas também pela elasticidade, pelo diâmetro da artéria e pela onda refletida.^16^ À luz desses dados, a pressão diastólica central é maior que a pressão diastólica periférica, e a pressão sistólica central é menor que a pressão sistólica periférica devido à onda refletida e à diferença nos diâmetros arteriais, respectivamente. O padrão ouro para o cálculo da PAM é obtido a partir da área sob a curva da forma de onda pressão-tempo de um ciclo cardíaco inteiro, e esse cálculo depende da integral ponderada no tempo das pressões intra-aórticas instantâneas.^1^ Para calcular a PAM com simplicidade, os pesquisadores usaram as PAS e PAD intra-aórticas com diferenças de tempo constantes. Nos estudos seguintes, a frequência cardíaca foi adicionada às fórmulas para obter valores de PAM mais corretos. Normalmente, a PAD é maior que a pressão sistólica. No entanto, na frequência cardíaca mais alta, o encurtamento do tempo diastólico é muito maior que o tempo sistólico. Como mencionado anteriormente, a onda refletida aumenta os valores da pressão diastólica e sistólica na aorta central; no entanto, o aumento diastólico é muito maior devido à duração do tempo diastólico. Portanto, o cálculo da PAM de acordo com a medição pontual das pressões arteriais (sistólica e diastólica) necessitou de ajustes para a frequência cardíaca. No entanto, na área periférica, a onda refletida é menos eficaz porque esses pontos estão mais próximos dos locais de reflexão, e a onda refletida precisa percorrer uma distância menor de volta. Portanto, o cálculo da PAM com base nas medidas do manguito do esfigmomanômetro não necessita de correção da dependência temporal, de modo que a fórmula corrigida inclui apenas os valores de PP e PAD. Em nosso estudo, o desvio do coeficiente de pressão de pulso da fórmula padrão apresentou a correlação mais forte com a PAD (R = -0,393, p < 0,001) em comparação com outras variáveis, como PAS (R = -0,285, p < 0,001) e idade (R = -0,174, p < 0,035).

O aumento da PAD medida pelo manguito é maior do que o aumento da PAD central (Figura 1). Essa situação pode estar relacionada a dois mecanismos diferentes. Primeiro, a função elástica da aorta é maior do que a das artérias periféricas.^17^ Portanto, em algum nível, a PAD mais alta não pode ser compensada pelas artérias periféricas, ao contrário da aorta. Em segundo lugar, a medição intra-aórtica é obtida diretamente por um cateter de angiografia. No entanto, a medição do manguito é obtida do lado de fora da artéria e está associada à pressão refletida na parede arterial. Portanto, o aumento da pressão arterial distende mais a parede arterial, resultando em artérias mais rígidas e aumento das medidas do manguito. Todos esses possíveis mecanismos podem explicar a discrepância entre as medições da PAD periférica e central. A diferença entre a pressão aórtica média medida e calculada é negativamente correlacionada com a pressão diastólica medida pelo manguito quando a fórmula padrão é usada. No entanto, a PAD mais alta medida pelo manguito não afeta a diferença entre a pressão aórtica média medida e a calculada quando a fórmula corrigida é utilizada (Figura 3). Isso nos mostra que a fórmula corrigida é mais confiável em valores mais altos de PAD, pois a fórmula corrigida possui uma correção da PAD no desvio do coeficiente de pressão de pulso.

### Limitações

Primeiro, as medidas do manguito foram realizadas a partir da artéria braquial; no entanto, não confirmamos o fluxo sanguíneo das artérias subclávia, axilar e braquial, e que qualquer grau de estenose pode afetar as medidas da pressão arterial. Segundo, as medidas do manguito foram obtidas enquanto o paciente estava deitado e o manguito estava no mesmo nível do coração. No entanto, na prática clínica, as medidas do manguito são realizadas quando os pacientes estão sentados e o nível do manguito é um pouco mais baixo em comparação com o coração. Isso também resulta em pressões mais altas do manguito. Finalmente, a rigidez arterial é afetada por algumas características clínicas, como idade, hipertensão e tabagismo. As medidas periféricas do manguito geralmente dependem da rigidez arterial. Portanto, os próximos estudos devem incluir pacientes com características clínicas semelhantes.

## Conclusão

Após a validação da fórmula corrigida nos próximos estudos, esta fórmula poderá ser testada em diferentes cenários clínicos, como choque séptico, insuficiência cardíaca aguda descompensada, comunicação interventricular aguda pós-infarto do miocárdio e ruptura de cordas. Em conclusão, nossa fórmula corrigida é superior à fórmula padrão para estimativa precisa da pressão arterial média aórtica. Todos os parâmetros de precisão utilizados neste estudo demonstram maior precisão da nova fórmula corrigida. Além disso, a fórmula padrão é mais propensa a erros de cálculo em valores mais altos de pressão arterial diastólica. No entanto, a fórmula corrigida funciona bem, independentemente do valor da pressão arterial diastólica, sistólica e da frequência cardíaca.
