# Supplementary material for: Uma Nova Fórmula Corrigida para Estimar Corretamente a Pressão Aórtica Central a Partir de Medições Periféricas da Braçadeira
Source: Arq Bras Cardiol. 2025 Sep 8;122(9):e20240880. [Article in Portuguese] doi: 10.36660/abc.20240880 (PMC12677885; doi:10.36660/abc.20240880)
Supplement: Translation

# Translation: A New Corrected Formula for Correct Estimation of Mean Central Aortic Pressure from Peripheral Cuff Measurements

**DOI:** 10.36660/abc.20240880i

**Authors:** Mehmet Ozgeyik, Onur Kaypakli

**Affiliations:** 1 Turquia Eskisehir City Hospital, Department of Cardiology, Eskisehir – Turquia; 2 Turquia Hatay University, Faculty of Medicine, Department of Cardiology, Hatay – Turquia

**Keywords:** Arterial Pressure, Pulso, Palpation

## Abstract

**Background:**

Mean arterial pressure (MAP) has critical importance in tissue perfusion. In clinical practice, the most used formula was suggested by Gauer, which uses systolic (SBP), diastolic (DBP), and pulse (PP) pressures gathered via the iliac artery (MAP = DBP + 0.333 x PP). However, its results are not reliable for noninvasive recordings as blood pressures are higher.

**Objectives:**

We derived a corrected formula for the correct calculation of MAP from the noninvasive cuff blood pressure recordings: MAP = DBPcuff + [0.33 + (0.43 – 0.0038 x DBPcuff)] x PPcuff.

**Methods:**

149 patients were included in this study. Intra-aortic and cuff blood pressure tracings were obtained simultaneously. The PP coefficient of the standard formula is 0.333 for all calculations. The PP coefficient deviation of the standard formula was calculated with the formula of PP coefficient − 0.333. These two formulas were compared using linear regression analysis and Akaike information criterion (AIC). The level of significance was set at 5% in the statistical analysis.

**Results:**

The measured intra-aortic mean pressure was 111.5±13.0 mmHg. The calculated intra-aortic mean pressure by the standard formula and the corrected formula was 105.8±13.5 and 111.3±12.1, respectively. The R, R^2^, and AIC of the corrected formula were better than the standard formula [(0.905 vs 0.887), (0.818 vs 0.787), and (858.9 vs 1002.7), respectively].

**Conclusion:**

To the best of our knowledge, this is the first study for the calculation of the MAP from cuff measurements, and the corrected formula has better accuracy than the standard formula for estimation of the MAP.

## Introduction

Tissue perfusion is very important to maintain the vital functions of the body. Mean arterial pressure (MAP) has critical importance in tissue perfusion. Both higher and lower values of MAP affect the cell mechanisms negatively. Therefore, the body should ensure a stable MAP, and the correct calculation of MAP is lifesaving in critical situations.

Calculation of the exact mean central blood pressure is gained from the intra-aortic pressure. The area under the curve of the pressure–time waveform of one entire cardiac cycle with the time-weighted integral is the correct way to calculate mean central blood pressure.^1^ Calculations of MAP by different formulas have been suggested so far. The first formula suggested by Gauer uses systolic (SBP), diastolic (DBP), and pulse (PP) pressures gathered via the iliac artery (MAP=DBP+0.333×PP) .^2^ In addition, the next studies showed that this formula had low accuracy, and they added parameters like heart rate (HR) to obtain more correct formulas.^3-5^ Lastly, Kaypakli et al. compared all formulas and suggested a better formula for the MAP calculation (MAP=0107×PP)+(−0.06+0.000773×HR)]×PP) .^6^ However, obtaining intra-aortic blood pressure values is not usually feasible in daily clinical practice.

In clinical practice, cuff sphygmomanometer blood pressure parameters are used for MAP calculation. Gauer’s formula is generally used for MAP calculation as it is easy to use. However, this empirical formula has not been compared to a better formula before. In this study, we derived a corrected formula for the calculation of mean central blood pressure from cuff blood pressure recordings:  Mean aortic  pressure = DBP cuff +[0.33+(0.43−0.0038× DBPcuff )]× PP cuff  .To the best of our knowledge, this is the first study for the calculation of the mean aortic pressure from cuff measurements.

## Materials and Methods

One hundred forty-nine patients (70 males, 79 females) who underwent elective coronary angiography were included in this study. *We included all patients who were compatible with the exclusion criteria between May 2023 and December 2023 (All-comers design). The sample size was not determined at the beginning of the study*. Patients with left ventricular ejection fraction < 50%, mild or severe valve diseases, electrolyte imbalance, rhythm disturbances, acute coronary syndromes, and younger than 18 years were excluded from the study. All patients provided written informed consent, and our Local Ethics Committee approved the study.

Medical history, blood parameters, and baseline characteristics were gained from hospital recordings. The same cardiologist performed echocardiography visualizations before the angiographies. Left ventricular ejection fraction was calculated by Simpson’s equation.

All coronary angiographies were performed via the femoral artery. 6 F diagnostic catheters (Pig-tail) were placed in the aortic root for calculation of the mean aortic pressures. MAP was computed by the area-under-the-pressure-time curve method. A standard automated oscillometric device (Bosomat, Bosooscillomat, Bosch, Jungingen, Germany, bladder size 28 x 12.5) was used for peripheral cuff blood pressure measurements. Intra-aortic and cuff blood pressure tracings were obtained simultaneously. During the measurements, all patients were in sinus rhythm. The same cardiology specialist obtained all measurements.

### Statistical analyses

The Kolmogorov-Smirnov test was performed to test if the variables were normally distributed, and a p-value >0.05 was defined as normally distributed data. Categorical and continuous data were expressed as a percentage (%) and mean ± standard deviation (SD), respectively. Pearson’s correlation was used to examine the relationship between continuous variables. IBM SPSS Statistics for Windows v. 23 was used for statistical analyses, and p-values <0.05 were considered statistically significant.

We calculated the PP coefficient for measured values with the formula of (measured MAP-DBP)/PP . As the PP coefficient of the standard formula is 0.333 for all calculations, the PP coefficient deviation of the standard formula was calculated with the formula of PP coefficient − 0.333. Then we determined the correlations of the PP coefficient deviation of the standard formula with clinical continuous variables. As we decided that the DBP from cuff measurements is correlated most with PP coefficient deviation of the standard formula (R: -,393, p< 0.001), we performed a scatter plot analysis of PP coefficient deviation of the standard formula with DBP from cuff measurements. In this scatter plot graphic, we gathered this equation:  PP coefficient deviation =0.43−0.0038× DBP cuff  .Afterwards, we added this equation to the original formula, which is MAP=DBP+0.33×PP. . Therefore, we concluded with this formula:  Mean aortic pressure = DBP cuff +[0.33+(0.43−0.0038× DBP cuff) ]× PP cuff  The differences between the measured MAP and the calculated MAP were determined at each measurement point and were used to evaluate the accuracy of the two different formulas: the standard formula (MAP=DBP+(0.33×PP) and the corrected formula ( MAorticP = DBP  cuff +[0.33+(0.43−0.0038× DBP cuff )]× PP cuff ) . First, we used the graphical method described by Bland and Altman.^7^ We calculated R, R2, mean square residuals (MSR = sum of square residuals/n), and the root mean square error (RMSE = √MSR) using linear regression analysis. All six necessary assumptions for using linear regression analysis were verified. As higher R and R2 values indicate greater accuracy, they indicate perfect theoretical agreement when they are equal to 1. Lower values of RMSE and MSR indicate greater accuracy; they indicate perfect theoretical agreement when they are equal to 0. We also obtained the Akaike information criterion (AIC) from generalized linear models using the formula AIC=N×ln⁡(RSS)+2P .^8^

The AIC gives a mathematical value for the assessment of different calculation methods. Lower values indicate better accuracy. We finally tested the accuracy of the four formulas using multivariate linear regression analysis.

## Results

There were a total of 298 blood pressure measurement points from 149 different patients (149 intra-aortic measurements and 149 simultaneous cuff measurements). The information regarding the baseline characteristics of the study population is shown in Table 1. The measured intra-aortic mean pressure was 111.5 ± 13.0 mmHg. The calculated intra-aortic mean pressure by the standard formula and the corrected formula was 105.8 ± 13.5 and 111.3 ± 12.1, respectively. Figure 1 shows the negative correlation between cuff-measured DBP and the difference between intra-aortic diastolic BP and cuff-measured DBP. This result shows us that cuff diastolic measurements increase more than aortic measurements at high diastolic values. The differences between measured and calculated mean aortic pressures as described by Bland and Altman^7^ for the standard formula and the corrected formula were demonstrated in Figure 2. Figure 3 shows the correlations between the differences between measured and calculated mean aortic pressure and cuff-measured DBP for standard and calculated formulas. This result points out that the corrected formula works more properly than the standard formula at higher DBP values (Central Illustration).

**Table 1:** – Baseline clinical and demographic features

Variables	N=149
Age (years)	55.2 ± 10.3
Sex Male,%)	70 (47.0)
Diabetes (n,%)	50 (33.6)
Hypertension (n,%)	87 (58.4)
Smoking status (n,%)	50 (33.6)
CAD history (n,%)	18 (12.1)
Hyperlipidemia (n,%)	47 (31.5)
Hemoglobin (g / dl)	13.8 ± 1.8
Creatinine (mg / dl)	0.83 ± 0.17
LDL (mg / dl)	111.9 ± 36.1
Intraaortic SBP (mmHg)	145.6 ± 22.7
Intraaortic DBP (mmHg)	86.6 ± 9.2
Cuff-measured SBP (mmHg)	143.9 ± 21.0
Cuff-measured DBP (mmHg)	86.7 ± 12.0
Cuff-measured Pulse pressure (mmHg)	57.2 ± 16.4
Measured intraaortic mean pressure (mmHg)	111.5± 13.0
Calculated intraaortic mean pressure (standard formula) (mmHg)	105.8 ± 13.5
Calculated intraaortic mean pressure (corrected formula)(mmHg)	111.3± 12.1
Heart rate (beat/min)	77.2 ± 11.8
Ejection Fraction	58.9± 3.6
Beta-blockers (n,%)	43 (28.9)
ACEi or ARB (n,%)	60 (40.3)
Calcium channel blocker (n,%)	25(16.8)
Diüretik (n,%)	44 (29.5)

LDL: low density lipoprotein; ACEi: angiotensin converting enzyme inhibitors; ARB: angiotensin receptor blockers; CAD: coronary artery disease; SBP: systolic blood pressure; DBP: diastolic blood pressure.

**Figure 1:** – Correlation between cuff-measured diastolic BP and the difference between intra-aortic diastolic BP and cuff-measured diastolic BP.

**Figure 2:** – Differences between measured and calculated mean aortic pressures according to heart rate for the standard formula and the corrected formula.

**Figure 3:** – Correlation between the differences between measured and calculated mean aortic pressure and cuff-measured diastolic blood pressures for standard and calculated formulas.

Bivariate correlation analysis of clinical continuous variables with pulse pressure coefficient deviation of the standard formula is shown in Table 2. In addition, Figure 4 demonstrates a positive correlation between the PP coefficient deviation of the standard formula and cuff-measured DBP.

**Table 2:** – Bivariate correlation analysis of clinical continuous variables with pulse pressure coefficient deviation of the standard formula

Variables	r	p
Heart rate	-0,079	0.337
Systolic blood pressure	-0,285	< 0.001
Diastolic blood pressure	-0,393	< 0.001
Pulse pressure	-0,078	0.344
Age	-0,174	0.035
Ejection Fraction	0,048	0.579

Pulse pressure coefficient: (Intra-aortic mean arterial pressure - cuff diastolic blood pressure)/ cuff Pulse pressure. Pulse pressure coefficient deviation of the standard formula: Pulse pressure coefficient - 0.33.

**Figure 4:** – Correlation between PP coefficient deviation of the standard formula and cuff-measured diastolic blood pressure.

Parameters of accuracy to predict mean central blood pressure are shown in Table 3. Although higher values of R and R^2^indicate greater accuracy, lower values of RMSE and MSR show greater accuracy. The R of the corrected formula (0.905) was better than the standard formula (0.887). The R^2^ of the corrected formula (0.818) was better than the standard formula (0.787) (Figure 5). The RMSE and MSR of the corrected formula were 5.577 mmHg and 31.104 mmHg^2^, respectively, which were better than the standard formula, which was 6.042 mmHg and 36.506 mmHg^2^, respectively. Lower AIC values indicate better accuracy. The AIC value of the corrected formula (858.9) was superior to the standard formula (1002.7).

**Table 3:** – Comparison of accuracy parameters of different formulas to predict mean arterial pressure

Accuracy parameter	Standard formula	Corrected Formula
R	0.887	0.905
R^2^	0.787	0.818
Mean square residuals (mmHg^2^)	36.506	31.104
Root mean square error (mmHg)	6.042	5.577
Akaike information criterion (AIC)	1002.746	858.912

**Figure 5:** – Comparison of intra-aortic mean blood pressure of standard formula and corrected formula according to R and R2.

Measured mean central blood pressure was independently predicted by only the corrected formula in the multivariate linear regression analysis (beta = 0.975, p < 0.001). Table 4 shows the multivariate linear regression analysis.

**Table 4:** – Multivariate linear regression analysis of two different formulas to predict the measured mean aortic pressure

	Beta	95% CI for Beta	p
Corrected Formula	0.975	0.901 -1.050	<0.001
Standard Formula	0.097	-0.205 - 0.398	0.528

## Discussion

To the best of our knowledge, this is the first study for the calculation of the mean aortic pressure from cuff measurements. The main finding of this study is that when compared to the standard formula of Gauer,^2^ the new corrected formula was found to be more accurate than the standard formula in terms of all accuracy criteria.

Increased arterial blood pressure is related to cardiovascular morbidity and mortality.^9^ Especially, central aortic pressure (CAP) predicts cardiovascular events more than peripheral blood pressure.^10^ In contrast to increased CAP, in the situation of sepsis and shock, MAP has a critical importance for estimating end-organ damage.^11,12^ MAP is targeted to be above 65 mmHg to reduce organ failure for septic shock; however, the calculation depends on central or radial arterial cannulation. Therefore, the estimation of CAP with noninvasive techniques may be very beneficial for clinicians in terms of the decision-making process, especially to predict organ perfusion in patients with critical conditions such as aortic dissection, sepsis, and shock.^13-15^ However, CAP calculation is more difficult and expensive than cuff measurements and needs an invasive approach. Many formulas have been created to find out the most accurate MAP calculation.^2-6^ However, these formulas were produced using the values of the CAP. To the best of our knowledge, this is the first study for the calculation of the mean aortic pressure from cuff measurements.

The corrected formula is compared with the standard formula. The corrected formula is superior to the standard formula according to all accuracy parameters of AIC, R, R^2^, MSR, and RMSE. In multivariate linear regression analysis, the new corrected formula predicts mean aortic pressure independently, unlike the standard formula. All these statistical analyses showed that the corrected formula calculates mean aortic pressure more accurately than the standard formula.

Peripheral artery pressure is affected by not only cardiac stroke volume, but also elasticity, the diameter of the artery, and the reflected wave.^16^ In light of these data, central diastolic pressure is more than peripheral diastolic pressure, and central systolic pressure is less than peripheral systolic pressure because of the reflected wave and the difference in arterial diameters, respectively. The gold standard for MAP calculation is obtained from the area under the curve of the pressure–time waveform of one entire cardiac cycle, and this calculation depends on the time-weighted integral of the instantaneous intra-aortic pressures.^1^ In order to calculate MAP with simplicity, researchers used intra-aortic systolic and diastolic pressures with constant time differences. In the next studies, heart rate was added to formulas to obtain more correct MAP values. Normally, DBP is longer than systolic pressure. However, in the higher heart rate, diastolic time shortening is much more than systolic time. As mentioned before, reflected wave augments the SBP and DBP values in the central aorta; however, diastolic augmentation is much greater because of diastolic time duration. Therefore, the calculation of the MAP according to spot measurement of SBP and DBP needed adjustments for heart rate. However, in the peripheral area, the reflected wave is less effective because these points are closer to the reflection sites, and the reflected wave has to travel back a shorter distance. Therefore, MAP calculation according to sphygmomanometry cuff measurements does not need time dependency correction, so the corrected formula includes only PP and DBP values. In our study, the pulse pressure coefficient deviation of the standard formula was found to be most strongly correlated with DBP (R = −0.393, p < 0.001) compared with other variables such as SBP (R = −0.285, p < 0.001) and age (R = -0.174, p < 0.035).

The increase in cuff-measured DBP is greater than the increase in central DBP (Figure 1). This situation may be related to two different mechanisms. Firstly, the elastic function of the aorta is greater than peripheral arteries.^17^ Therefore, at some level, higher diastolic blood pressure cannot be compensated by the peripheral arteries, unlike the aorta. Secondly, intra-aortic measurement is obtained directly by an angiography catheter. However, cuff measurement is obtained from the outside of the artery and is associated with the pressure reflected on the arterial wall. So, increased blood pressure stretches the artery wall more, resulting in stiffer arteries and increased cuff measures. All of these possible mechanisms may explain the discrepancy between the peripheral and central DBP measurements. The difference between measured and calculated mean aortic pressure is negatively correlated with the cuff-measured diastolic pressure when the standard formula is used. However, higher cuff-measured DBP does not affect the difference between measured and calculated mean aortic pressure when the corrected formula is used (Figure 3). This shows us that, corrected formula is more reliable in higher DBP values because the corrected formula has a DBP correction in pulse pressure coefficient deviation.

### Limitations

First, cuff measurements were performed from the brachial artery; however, we did not confirm the blood flow of the subclavian, axillary, and brachial arteries, and that any degree of stenosis may affect the blood pressure measurements. Second, the cuff measurements were obtained while the patient was lying down and the cuff was at the same level as the heart. However, in clinical practice, cuff measurements are performed when the patients are sitting down and the cuff level is a bit lower compared with the heart. This also results in higher cuff pressures. Finally, arterial stiffness is affected by some clinical features like age, hypertension, and smoking. Peripheral cuff measurements usually depend on arterial stiffness. Therefore, the next studies should include patients with similar clinical features.

## Conclusion

After validation of the corrected formula in the next studies, this formula may be tested with different clinical scenarios such as septic shock, acute decompensated heart failure, acute post-myocardial infarction-related ventricular septal defect, and chordal rupture. In conclusion, our corrected formula is superior to the standard formula for accurate estimation of aortic mean arterial pressure. All of the accuracy parameters used in this study show better accuracy of the new corrected formula. In addition, the standard formula is more prone to a miscalculation in higher diastolic blood pressure values. However, the corrected formula works well, independently of the value of the diastolic, systolic blood pressure, and heart rate.
